# Correcting Hypokalemia in Hospitalized Patients Does Not Decrease Risk of Cardiac Arrhythmias

**DOI:** 10.1155/2019/4919707

**Published:** 2019-09-24

**Authors:** Weston Harkness, Paula Watts, Michael Kopstein, Oliwier Dziadkowiec, Gregory Hicks, Dmitriy Scherbak

**Affiliations:** ^1^Graduate Medical Education, Sky Ridge Medical Center, Lone Tree, Colorado, USA; ^2^Graduate Medical Education, Rocky Vista Health Center, Parker, Colorado, USA; ^3^Graduate Medical Education, Continental Division of HCA, Denver, Colorado, USA

## Abstract

**Background:**

It is currently standard practice to correct hypokalemia for the purpose of preventing cardiac arrhythmias in all hospitalized patients. However, the efficacy of this intervention has never been previously studied.

**Objective:**

The objective of our study was to evaluate whether patients without acute coronary syndrome or history of arrhythmias were at increased risk of clinically significant cardiac arrhythmias if their potassium level was not corrected to ≥3.5 mEq/L.

**Design:**

A retrospective case control study.

**Setting:**

A community hospital.

**Participants:**

We enrolled selected patients who had episodes of hypokalemia during their hospital stay and were monitored on telemetry. Patients were split into groups based on success of replacing serum potassium to ≥3.5 mEq/L after 24 hours.

**Measurements:**

The primary outcome was the development of an arrhythmia. Arrhythmias included supraventricular tachycardia, atrial fibrillation, atrial flutter, Mobitz type II second-degree or third-degree AV block, ventricular tachycardia, or ventricular fibrillation. A one-tailed Fisher's exact test and logistic regression were used for analysis.

**Results:**

A total of 1338 hypokalemic patient days were recorded. Out of these days, 22 arrhythmia events (1.6% of patient days) were observed, 8 in the uncorrected group (1% patient days) and 14 in the corrected group (2.6% patient days). We found no statistically significant relationship between successfully correcting potassium to ≥3.5 mEq/L and number of arrhythmic events (*p*=0.037, OR = 2.38 (95% CI: 0.99, 6.03)). Logistic regression revealed that correction of potassium does not seem to be significantly related to arrhythmias (*β* = 0.869, *p*=0.0517).

**Conclusions:**

In the acute care setting, we found that patients with hypokalemia whose potassium level did not correct to ≥3.5 mEq/L were not at increased odds of having an arrhythmia. This study suggests that the common practice of checking and replacing potassium is likely inconsequential.

## 1. Introduction

Hypokalemia (serum potassium level less than 3.5 mEq/L) is a common electrolyte abnormality found in as many as 20% of hospitalized patients [1]. Potassium concentration is regulated by the kidney and is important in a number of physiologic processes such as maintenance of smooth muscle tone, cardiac electrical conduction, and acid-base balance [2]. In hospitalized patients it is common to routinely monitor serum potassium levels and administer potassium supplementation via oral or parenteral routes to maintain serum concentrations at 3.5 mEq/L or above. It is believed that patients with severe hypokalemia demonstrate specific electrocardiographic changes that lead to arrhythmias [3]. However, evidence for potassium replacement is largely based on animal studies that have shown increased incidence of arrhythmias with severe hypokalemia [4]. There is also retrospective evidence that hypokalemia leads to an increase in arrhythmias in the setting of acute myocardial infarction [5]. In patients without acute coronary syndrome or history of arrhythmia, there have been no identified studies that associate correcting hypokalemia with decreasing the risk of clinically significant arrhythmias.

Because hypokalemia is such a common finding even in the noncardiac population and results in significant expense and use of hospital resources, there is a need to better understand the risk of developing clinically significant arrhythmias in these patients. Our objective was to evaluate whether patients without acute coronary syndrome or history of arrhythmias are at increased risk of clinically significant cardiac arrhythmias if their potassium level is not corrected to ≥3.5 mEq/L.

## 2. Methods

After institutional review board exemption status was obtained, we conducted a retrospective case-control study of patients who were admitted to a community medical center from January 2015 to January of 2017. Patients aged 19 to 89 who were admitted and monitored on telemetry who were diagnosed with or developed hypokalemia during their hospital stay were identified by International Classification of Diseases diagnosis codes recorded in the electronic medical record. At-risk patient groups such as pregnant females and prisoners were excluded.

We attempted to exclude major risk factors such as history of arrhythmias and ischemic heart disease. Therefore, patients with a personal history of atrial fibrillation, atrial flutter, ventricular tachycardia, ventricular fibrillation, or Wolff–Parkinson–White syndrome were excluded. Patients admitted for acute myocardial infarction, diabetic ketoacidosis, hyperglycemic hyperosmolar state, supraventricular tachycardia, atrial fibrillation, atrial flutter, ventricular tachycardia, ventricular fibrillation, and Mobitz type II second-degree or third-degree atrioventricular block were also excluded. A total of 530 patients were identified as eligible who were admitted for a total of 1338 hypokalemic patient days, each representing a 24-hour period of hypokalemia. Many patients had recurrent episodes of hypokalemia and therefore contributed to multiple data points. Patients were excluded from recording future hypokalemic days if an arrhythmia was observed. Demographics of the patients including age, sex, history of heart failure with reduced ejection fraction (HFrEF), and rhythm on admission were recorded.

For patients who met the inclusion and exclusion criteria, the medical record was analyzed retrospectively. Potassium level and subsequent potassium level at 24 hours were recorded. Patients were then separated into two groups based on whether potassium was successfully or unsuccessfully corrected to above 3.5 mEq/L after 24 hours. The route (oral or intravenous) and amount of replacement of potassium given was subject to physician discretion. For intravenous preparations, 10 mEq of potassium dissolved in 100 ml of NS was infused over 1 hour. For oral preparations, immediate release or extended release formulations were used at the ordering physician's discretion. During this hypokalemic patient day (24-hour time period during which the patient had hypokalemia), physician documentation was reviewed for any noted arrhythmias including supraventricular tachycardia, atrial fibrillation, atrial flutter, Mobitz type 2 second-degree or third-degree atrioventricular block, ventricular tachycardia, or ventricular fibrillation. The primary outcome of the study was the development of one of these arrhythmias.

## 3. Power Analysis

Power analysis for Fisher's exact test was performed based on our primary hypothesis.

Since the hypothesis was one tailed, we performed a one-tailed power analysis, assuming a power of 0.8, alpha of 0.05, and a 2% proportion difference between the two groups. This resulted in a total sample size requirement of 1,332 data points. Thus, our final sample size of 1338 was adequate [6].

## 4. Statistical Analysis

A one-tailed Fisher's exact test and logistic regression were used to evaluate the association between successful potassium replacement and cardiac arrhythmias in patients without acute coronary syndrome or history of arrhythmias. Additionally, an odds ratio (OR) was also calculated. In order to minimize confounding causes of arrhythmias, a multivariable analysis using logistic regression was controlled with HFrEF. Additionally, a Mann–Whitney *U* test was used to evaluate the association between amount of potassium replacement and cardiac arrhythmias. To compare demographics of the study population, a two-sided Person *X* [2] was used for sex and history of HFrEF. An independent sample *T* test was used to compare age. A Welch's *T*-test was used to compare initial potassium and a Mann–Whitney *U* test was used to compare amount of potassium received. Findings were considered significant at *p* < 0.05. All analysis was performed using SPSS 24 and Microsoft Excel.

## 5. Results

A total of 1338 hypokalemic patient days were recorded, representing 530 individual patients. 571 hypokalemic days were recorded in the corrected group and 767 hypokalemic days in the uncorrected group. Both the corrected and uncorrected groups were demographically similar with regard to sex or history of HFrEF. The uncorrected group was slightly but significantly older. The corrected group had a slightly but significantly higher initial potassium level and received more potassium (Table 1).

**Figure 1:**
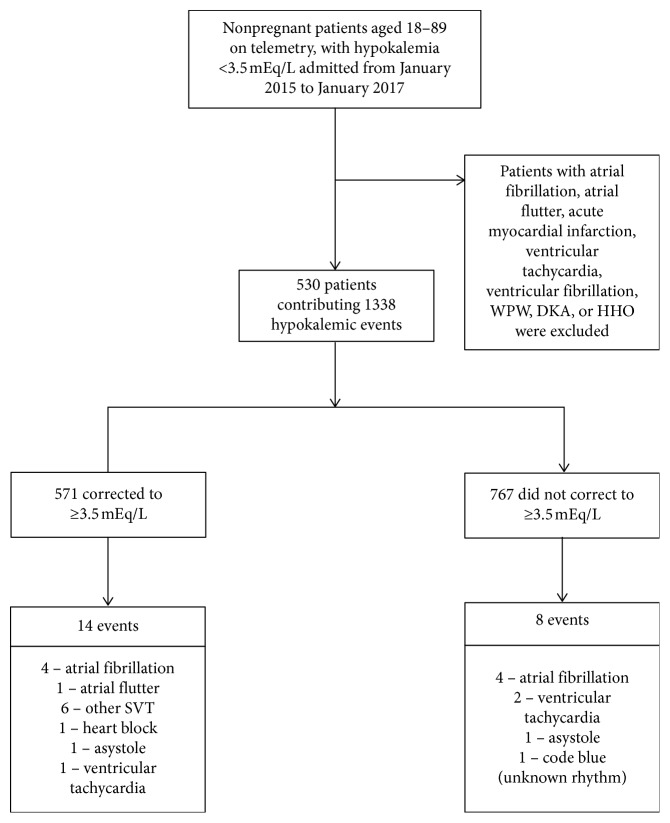
Study cohort creation along with number of patients in each group with number and type of each arrhythmia. WPW = Wolff–Parkinson–White syndrome; DKA = diabetic ketoacidosis; HHO = hyperglycemic hyperosmolar state; SVT = supraventricular tachycardia.

There were 22 arrhythmia events (1.6% patient days) observed, 8 in the uncorrected group (1% patient days) and 14 in the corrected group (2.6% patient days) (Figure 1). Using the one-tailed Fisher's exact test, we found no statistically significant relationship between correcting potassium to ≥3.5 mEq/L and number of arrhythmic events (*p* = 0.037, OR = 2.38 (95% CI: 0.99, 6.03)). Logistic regression also revealed that correction of potassium does not seem to be significantly related to arrhythmia (*β* = 0.869, *p* = 0.0517). When trying to control correction of potassium with HFrEF in predicting arrhythmias, the *β* and *p* values remain virtually the same and still not significant (*β* = 0.861, *p* = 0.0542); thus, HFrEF is likely not a confounder of this relationship between correcting potassium and arrhythmias. There is a statistically significant relationship between amount of potassium replaced and event of an arrhythmia (*W* = 9746.5, *p* = 0.008), as analyzed with the Mann–Whitney *U* test.

## 6. Discussion

Previous studies have provided evidence that hypokalemia increases the incidence of arrhythmias including atrial fibrillation in certain cardiac patients such as those with an acute ST-elevation myocardial infarction (STEMI) or those undergoing percutaneous coronary intervention [5, 7]. To the best of our knowledge, this retrospective study is the only analysis of the effect of potassium replacement on hypokalemic patients without acute coronary syndrome or history of arrhythmias. Of the 1338 recorded hypokalemic patient days, unsuccessful potassium replacement did not have increased odds of an arrhythmia during the hospital course. Subsequent logistic regression analysis showed that successful potassium replacement is not significantly related to arrhythmias. Interestingly, we found that the group which had an arrhythmic event received a statistically significant higher amount of potassium.

It is currently common practice to correct a patient's potassium level in the setting of hypokalemia due to the perceived risk of arrhythmia. This treatment can often be burdensome to patients due to the large size of potassium supplement pills, unpleasant taste of potassium packets, and discomfort associated with IV infusions [8]. Correction also leads to increased frequency of blood draws, increasing the cost of hospitalization with potentially minimal effect on patient outcomes [9]. The wholesale price of potassium supplements can range from $0.50 for a 20 mEq potassium chloride tablet up to $14.00 for a 15 mmol vial of potassium phosphate. These amounts quickly add up to significant cost burden to the patient and facility over days of repeated monitoring and replacement. Our study suggests that this burden and expense may be futile in our patient population. Even though there was a statistically significant relationship between amount of the potassium replaced and arrhythmic event, this result is difficult to interpret as the temporal relationship between arrhythmia and potassium replacement is unknown. It is plausible that patients had an arrhythmic event prior to potassium administration and thus were likely to receive more potassium.

This study was limited by the retrospective analysis performed at a single center which may not adequately represent a larger population. The study was also limited by low total number of observed arrhythmias in our study group. We were not able to exclude all risk factors for arrhythmias, thus potentially including confounding variables. A selection bias may also exist as it was found that the uncorrected group had a statistically lower initial potassium and received less potassium than the corrected group. The study was also limited by a lack of a standardized protocol for potassium replacement. At the community center where the study was performed, only the intensive care unit has a standardized protocol. For the majority of subjects, the route and amount of potassium replacement were at the ordering physician's discretion. It may be informative to repeat this analysis on a larger population to capture more arrhythmias. A prospective study design may also be warranted to avoid documentation issues and limitations inherent to retrospective review.

In conclusion, this retrospective chart review of hypokalemia in patients without acute coronary syndrome or history of arrhythmias did not reveal increased odds of arrhythmia in patients whose potassium did not correct to ≥3.5 mEq/L. This evidence suggests that the common practice of potassium replacement in all hospitalized patients may be unwarranted.

## Figures and Tables

**Table 1:** Characteristics of the study cohort with associated *p* values for each characteristic.

	All	Uncorrected	Corrected	*p* value
Average age	62.92	63.75	61.80	0.02
Male	519	206	313	0.09
History of HFrEF	53	24	29	0.08
Average initial potassium (mEq/L)	3.09	3.07	3.12	0.000
Average potassium after 24 hrs (mEq/L)	3.42	3.12	3.82	
Average amount received (mEq)	50.36	45.83	56.46	0.000
Number of arrhythmias	22	8	14	

## Data Availability

The data that support the findings of this study are available from HCA HealthONE, but restrictions apply to the availability of these data which were used under license for the current study and so are not publicly available.
